# Penile trauma management in absence of fracture: Long‐term outcomes

**DOI:** 10.1002/bco2.70092

**Published:** 2025-09-28

**Authors:** Kalpesh Parmar, Anshu Jha, Angel John, Aditya Manjunath, Odunayo Kalejaiye, Ali Reza Vosough, Bhaskar Somani, Joe Philip

**Affiliations:** ^1^ Freeman Hospital Newcastle UK; ^2^ Bristol Urological Institute Southmead Hospital Bristol UK; ^3^ Southmead Hospital Bristol UK; ^4^ University Hospital Southampton NHS Foundation Trust Southampton UK; ^5^ Bristol Urological Institute, Southmead Hospital, Bristol, & Honorary Associate Professor, School of Engineering Mathematics and Technology University of Bristol UK

**Keywords:** andrology, conservative management, erectile dysfunction, imaging, penile fracture, surgery

## Abstract

**Objective:**

This study evaluates the clinical outcomes of patients with suspected penile fractures who were managed conservatively after MRI excluded tunica albuginea rupture or fracture.

**Methods:**

A retrospective review was conducted over a seven‐year period, identifying patients who presented with symptoms suggestive of penile fracture. All patients who underwent MRI imaging to confirm or exclude the presence of a tunica albuginea rupture. Based on MRI findings, patients without confirmed fractures were managed conservatively, including instructions to avoid sexual activity and strenuous physical exertion. Follow‐up assessments were conducted to monitor long‐term complications, with a specific focus on erectile function, assessed via the International Index of Erectile Function (IIEF) and penile curvature.

**Results:**

Of the 30 patients with suspected penile fractures, MRI excluded fractures in 63%. Among these conservatively managed patients, approximately 60% developed erectile dysfunction (ED) and 27% developed penile curvature. Even in cases without confirmed fractures, patients with contusions demonstrated significant post‐injury complications.

**Conclusion:**

MRI is effective in ruling out penile fractures, supporting the use of conservative management when fractures are not confirmed. However, conservative treatment alone is associated with a notable rate of complications, suggesting the potential benefit of early penile rehabilitation to address functional outcomes in these patients.

## 1 INTRODUCTION

Penile fractures, though rare, are considered urological emergencies that demand prompt diagnosis and intervention. They typically occur during sexual activity or physical trauma, when excessive force is applied to an erect penis. This results in a rupture of the tunica albuginea, the thick fibrous layer surrounding the corpora cavernosa.^1^ Patients often report a “snap” sensation, accompanied by immediate pain, detumescence and bruising of the penis. Approximately one‐third of patients may present with associated urethral injuries. These injuries are typically suspected in cases where patients exhibit haematuria and/or experience difficulty or inability to void.^2^ Rapid surgical repair has traditionally been the preferred treatment approach, as delayed treatment can result in long‐term complications such as erectile dysfunction (ED), penile curvature and fibrosis.

Diagnosing a penile fracture is commonly based on clinical evaluation, which includes patient history and physical examination. However, distinguishing between actual fractures and other forms of penile trauma, such as contusions, can be challenging. Magnetic resonance imaging (MRI) has emerged as a valuable diagnostic tool in cases where clinical assessment alone is inconclusive.^3^ MRI can provide detailed images of the penile structure, allowing for precise identification of injuries to the tunica albuginea, corpus cavernosum and associated structures. Studies have shown that MRI has a higher sensitivity than Doppler ultrasound (DUS) in detecting tunical tears, especially in emergency settings where accurate imaging is essential.^4^

This study aims to assess the clinical outcomes of patients with suspected penile fractures who were managed conservatively after MRI confirmed the absence of a fracture. By evaluating erectile function, penile curvature and other complications in these patients, this study provides insight into the potential benefits and limitations of conservative management following MRI evaluation.

## 2 METHODS

### 2.1 Study design and population

**FIGURE 1:**
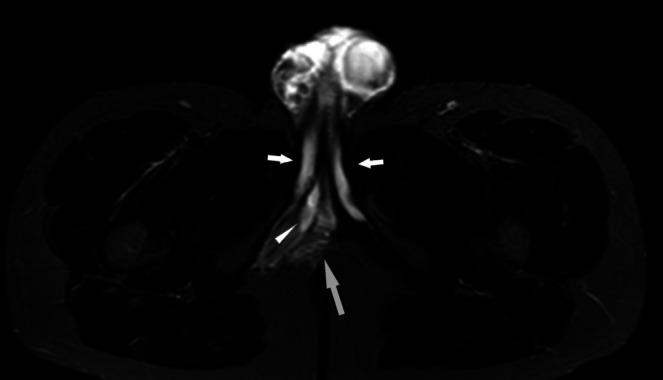
Axial STIR sequence MRI image of the perineum including the base of the penis. The two white tubular structures on each side are the corpora cavernosa (white arrows). The middle structure, which lies on the right side of the midline is the haematoma (white arrowhead), which together with the oedema (grey arrow) implies injury to the right corpus cavernosum, not shown on this image.

**FIGURE 2:**
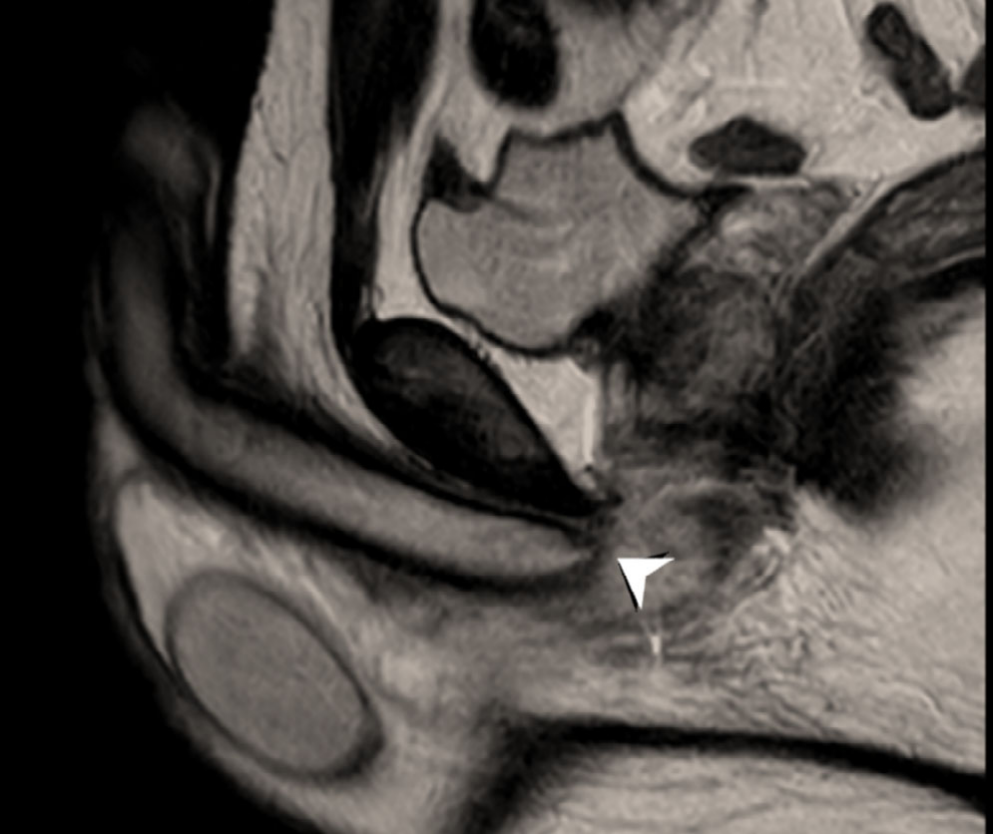
Sagittal T2‐weighted MRI image of the penis shows a small beak‐like protrusion (white arrowhead) near the base of the right corpus cavernosum in keeping with a small tear.

This retrospective study was conducted over seven years, from April 2018 to March 2024, at a tertiary urology centre. The study included male patients who presented with suspected penile fractures and underwent MRI evaluation. Only those with MRI findings that confirmed the absence of a penile fracture were included [Figures 1 and 2]. Patients who had primary surgical exploration due to confirmed clinical diagnoses or who were treated without MRI imaging were excluded.

### 2.2 Data collection

Demographic data, clinical presentation, cause of injury and MRI findings were collected for each patient. Injury causes were categorized into sexual trauma, which included sexual misadventure and specific practices such as *taqaandan* (a self‐induced penile manipulation), and non‐sexual trauma.

Follow‐up data were collected to assess clinical outcomes, including erectile dysfunction, penile curvature and other complications. Erectile dysfunction was evaluated using patient‐reported outcomes, and penile curvature was assessed based on visual inspection or patient complaints.

### 2.3 Statistical analysis

Descriptive statistics were used to analyse demographic information, injury causes, MRI findings and outcomes. Outcomes were compared among patients with MRI‐confirmed absence of fractures, including those with minor injuries or contusions.

## 3 RESULTS

### 3.1 Patient demographics and injury causes

A total of 30 men met the inclusion criteria, with an average age of 40.4 years (range: 22–65 years). The majority of injuries (63%) resulted from sexual trauma, including five patients (16%) who reported practicing *taqaandan*, while the remaining injuries (27%) were due to non‐sexual trauma [Table 1].

**TABLE 1:** Demographics of patients.

Parameter	Mean (SD) or %
Age	40.4 years (Range: 22–65)
Injury Cause	
‐ Sexual Trauma	93%
‐ Taqaandan Practice	17%
‐ Non‐Sexual Trauma	7%
MRI findings	
‐ No Fracture	19 (63%)
‐ Tunica Thinning or Scarring	5 (17%)
Cavernosal Injury without Fracture	6 (20%)

Mode of injury in almost all (93%) was injury during vigorous intercourse, but only 40% of these reported the expected ‘felt pop and detumescence’. The remaining attended with penile swelling and some degree of bruising. Two reported falling on the handlebars while cycling, and another an associated skateboard injury. Clinical examinations in this cohort did not show extensive bruising or a defect.

### 3.2 MRI findings

On MRI, 63% of patients (19/30) showed no evidence of fracture, whereas 37% (11 patients) exhibited signs of tunica thinning, cavernosal injury or scar tissue without a full‐thickness rupture [Table 1].

### 3.3 Clinical outcomes

Despite conservative management, 18 patients (60%) developed erectile dysfunction, of whom 7 had PDE5I and 3 had MUSE. Eight patients (27%) experienced penile curvature, with only one patient requiring surgical correction. Of those with ED, only one patient had an MRI‐confirmed penile contusion, while the others showed no detectable abnormalities on imaging [Table 2].

**TABLE 2:** Follow up outcomes of patients managed conservatively.

Outcome	Number
Erectile Dysfunction (ED)	18
Penile Deviation	8
Penile Plaque	5
Persistent Pain	3
No Complications	9

## 4 DISCUSSION

Our findings underscore MRI's critical role in assessing penile trauma and differentiating true fractures from other non‐surgical injuries. MRI's high sensitivity in detecting tunica thinning, scarring and cavernosal injuries makes it invaluable in clinical decision‐making. This study aligns with previous work by Stempel et al, who reported MRI's efficacy in identifying injuries that are equivocal on ultrasound and have unusual location of injury.^5^ Studies, including Spiesecke et al^6^ and Saglam et al,^7^ emphasize MRI's superiority over ultrasound for diagnosing penile trauma, noting its value in emergency urology settings for rapid and accurate assessment.

This accuracy allows clinicians to avoid unnecessary surgery in cases of MRI‐negative injuries, a finding that reflects the results reported by Panella et al,^8^ who found that MRI‐based conservative management was effective in over 30% of suspected penile fracture cases.

Despite MRI confirming the absence of fractures, long‐term complications were significant among our cohort, with 60% of patients developing erectile dysfunction (ED) and 27% experiencing penile curvature. This complication rate aligns with other conservative management studies, including research by Gamal et al,^9^ which found similar ED rates in non‐surgical cases of penile trauma. The prevalence of ED suggests that penile injuries may lead to microvascular damage or fibrosis, which can impair erectile function, as corroborated by Tsao et al^10^ and Penson et al^11^ in their study on trauma‐induced erectile impairment.

Notably, microvascular and cavernosal injuries, which MRI might not always detect, could contribute to these functional deficits. They argue that subclinical trauma can lead to fibrotic tissue changes that progressively worsen erectile quality over time, despite an initial MRI‐negative diagnosis.^12^

Compared to who undergo immediate surgical repair for confirmed fractures, those managed conservatively after MRI‐negative results appear to face a higher risk of long‐term complications. Studies by Yamacake et al support surgical intervention for confirmed fractures to prevent ED and penile curvature, citing complication rates as low as 10% when surgery is performed within 24 hours of injury.^13^

Conversely, delayed or conservative management has higher complication rates. Yapanaglu et al compared conservative and surgical treatment for penile fractures and documented an 80% rate of ED and curvature in cases where patients were not promptly surgically managed.^14^ In a large multi‐centre European study, men who underwent surgical repair after 8 hours from admission to the emergency department had significantly worse erectile function at 1‐ and 3‐month post‐operative.^15^ These findings suggest that while MRI‐guided conservative management avoids unnecessary surgeries, penile trauma in the absence of MRI fracture still carries risks for functional impairment, a factor that urologists should consider when advising patients on management options. A careful selection of uncomplicated penile fracture cases for conservative management can yield favourable outcomes. This was demonstrated in a study by Muentener et al., which included 29 cases of penile fractures, 59% of which were managed conservatively. With a mean follow‐up period of 67 months, only three patients in the conservatively treated group reported poor outcomes, while the majority showed satisfactory results during follow‐up.^16^ This study highlights the importance of patient selection and monitoring in achieving successful outcomes with conservative approaches to penile fractures.

**FIGURE 3:**
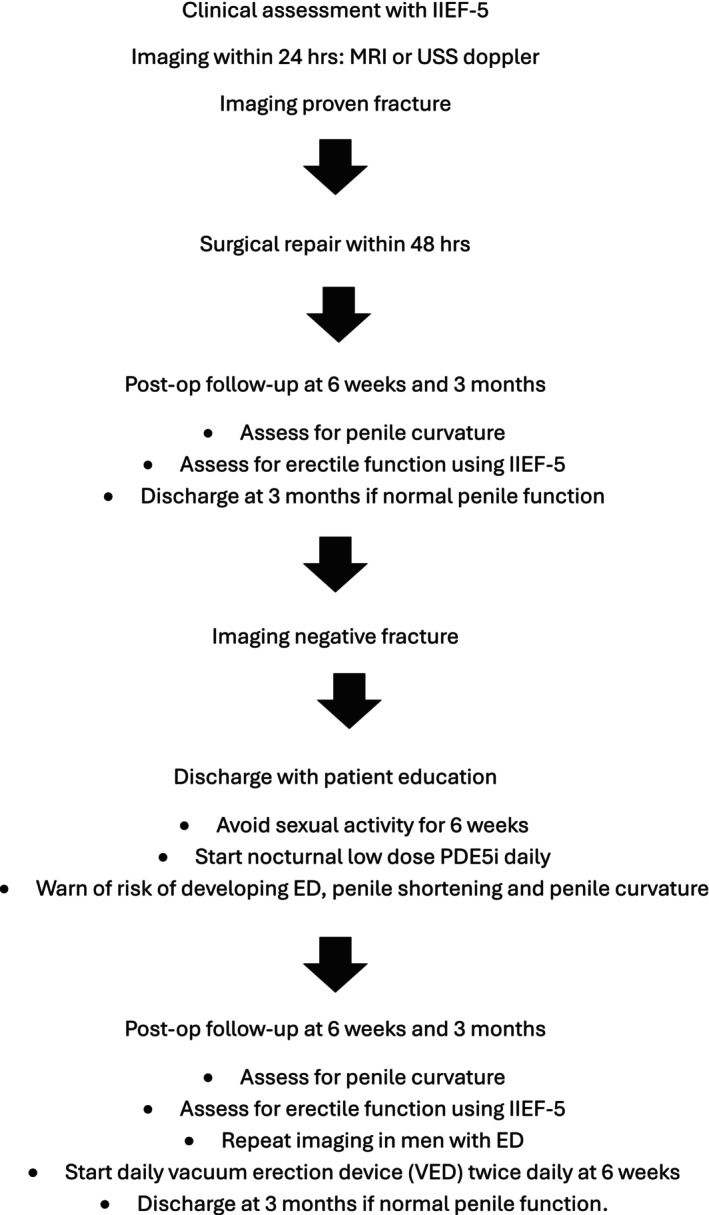
Protocol for penile trauma management.

Given the high incidence of ED and penile curvature observed in our MRI‐negative cohort, early penile rehabilitation may benefit these patients. Basal et al assessed an optimal penile rehabilitation programme on erectile function after radical prostatectomy and concluded that the use of phosphodiesterase type 5 inhibitors (PDE5Is) and vacuum erection devices has a beneficial effect on erectile function recovery across all levels of baseline erectile function.^17^ Applying similar interventions in patients with conservatively managed men without penile fracture theoretically help mitigate fibrosis and support erectile function [Figure 3].

Several limitations affect this study's generalizability. Our retrospective design and the relatively small sample size limit the strength of conclusions and call for more extensive, prospective studies to validate our findings. Although MRI is usually considered the gold standard for diagnosing Tunica rupture, this is not always practical in all centres. There are many centres where the use of USS doppler provides good results, especially where expertise has been developed in penile USS imaging or when the patient presents out of hours. Bozzini et al. had 60% diagnosed with USS doppler.^15^ Furthermore, the reliance on subjective self‐reporting for ED and curvature could introduce reporting bias. Future studies with objective assessments of erectile function and penile curvature would add valuable data on the outcomes of conservatively managed penile trauma.

There is a clear need for standardized protocols for managing MRI‐negative penile trauma. Berstein et al. recently reviewed the contemporary literature pertaining to optimal timing of penile fracture and emphasized the importance of early intervention to reduce complications and better functional outcomes, suggesting that conservative treatment alone may not suffice for long‐term sexual function preservation.^18^ Larger studies could help refine management strategies. We would suggest that perhaps men with penile trauma who are MRI negative should be reviewed at weeks to assess for penile curvature and ED. There are currently no guidelines with regards to repeating imaging when initial scans do not reveal a penile fracture. However, this may be an area for further research; repeat imaging may be implemented at 6 weeks in men with ED. The integration of early rehabilitation as a routine practice in penile injuries to minimize ED and curvature risks. While data specific to penile fractures are limited, the theoretical benefits of these therapies justify their consideration in clinical practice. Prospective studies investigating the role of adjunctive therapies in penile trauma are warranted to establish evidence‐based guidelines. Due to the rare nature of this condition, collaborative studies across different countries and utilizing national datasets may be required.

In conclusion, penile fractures represent a complex clinical challenge requiring prompt diagnosis and individualized management. While surgery remains the cornerstone of treatment, conservative management for MRI‐negative men may be appropriate for selected patients, provided they are closely monitored. The emergence of MRI as a diagnostic tool has revolutionized the evaluation of penile trauma, enabling clinicians to tailor treatment plans based on injury severity.

The potential for adjunctive therapies such as PDE5Is and VEDs to mitigate long‐term complications offers a promising avenue for improving outcomes. Future research should focus on refining management protocols and exploring the role of rehabilitation in penile trauma care. By embracing a multidisciplinary approach, clinicians can optimize functional outcomes and enhance the quality of life for patients with penile fractures.

## AUTHOR CONTRIBUTIONS

*Concept*: Kalpesh Parmar, Ali Reza Vosough, Bhaskar Somani, Joe Philip. *Design*: Kalpesh Parmar, Anshu Jha, Aditya Manjunath, Odunayo Kalejaiye, Ali Reza Vosough, Bhaskar Somani, Joe Philip. *Supervision*: Kalpesh Parmar, Anshu Jha, Aditya Manjunath, Odunayo Kalejaiye, Ali Reza Vosough, Bhaskar Somani, Joe Philip. *Resources*: Kalpesh Parmar, Anshu Jha, Angel John, Aditya Manjunath, Odunayo Kalejaiye, Ali Reza Vosough, Bhaskar Somani, Joe Philip. *Materials*: Kalpesh Parmar, Anshu Jha, Angel John, Aditya Manjunath, Odunayo Kalejaiye, Ali Reza Vosough, Bhaskar Somani, Joe Philip. *Data collection and/or processing*: Kalpesh Parmar, Anshu Jha, Angel John, Aditya Manjunath, Odunayo Kalejaiye, Ali Reza Vosough, Bhaskar Somani, Joe Philip. *Analysis and/or interpretation*: Kalpesh Parmar, Anshu Jha, Angel John, Aditya Manjunath, Odunayo Kalejaiye, Ali Reza Vosough, Bhaskar Somani, Joe Philip. *Literature search*: Kalpesh Parmar, Anshu Jha, Angel John, Aditya Manjunath, Odunayo Kalejaiye, Ali Reza Vosough, Bhaskar Somani, Joe Philip. *Writing manuscript*: Kalpesh Parmar, Anshu Jha, Angel John, Aditya Manjunath, Odunayo Kalejaiye, Ali Reza Vosough, Bhaskar Somani, Joe Philip. *Critical review*: Kalpesh Parmar, Anshu Jha, Angel John, Aditya Manjunath, Odunayo Kalejaiye, Bhaskar Somani, Joe Philip.

## CONFLICT OF INTEREST STATEMENT

All authors have reported no Competing Interest. The authors declare that they have not received support from any organization for the submitted work; no financial relationships with any organizations that might have an interest in the submitted work; and no other relationships or activities that could appear to have influenced the submitted work.
